# SiO_2_ Nanoparticles-Acrylate Formulations for Core and Cladding in Planar Optical Waveguides

**DOI:** 10.3390/nano11051210

**Published:** 2021-05-03

**Authors:** Leonid M. Goldenberg, Mathias Köhler, Christian Dreyer

**Affiliations:** 1 Department of Engineering and Natural Sciences, Technical University of Applied Sciences Wildau, Hochschulring 1, 15745 Wildau, Germany; 2 Research Division Polymeric Materials and Composites PYCO, Fraunhofer-Institute for Applied Polymer Research, Schmiedestraße 5, 15745 Wildau, Germany; mathias.koehler@iap.fraunhofer.de

**Keywords:** fluorinated acrylate, silica nanoparticles, nanocomposite, thermo-optic coefficient, optical propagation losses

## Abstract

A combination of acrylate formulations and SiO_2_ nanoparticles is investigated with the aim to improve the optical properties of low-refractive index polymers that are used for the fabrication of planar optical waveguides. A decrease in refractive index and also in the thermo-optic coefficient of nanocomposite materials is clearly demonstrated, while some formulations exhibit an increase in the glass transition temperature. The possibility of using these nanocomposite materials to fabricate waveguiding layers with low optical propagation losses at telecommunication wavelengths around 1550 nm is also shown. The nanomaterials can be applied in optical microchips on polymer platforms.

## 1. Introduction

Low-refractive index fluorinated polymers have found an application as passive waveguiding materials in planar optical waveguides [1,2,3,4,5,6,7,8] and optical fibers [9]. The main requirements for such materials are low-refractive indices (1.5 or smaller) and low optical propagation losses. The possibility of tuning the thermo-optic coefficient (TOC) is also important, while different applications (passive optical waveguides or thermo-optical switches) require different TOC. Materials for passive optical waveguides should exhibit a low TOC.

**Scheme 1:**
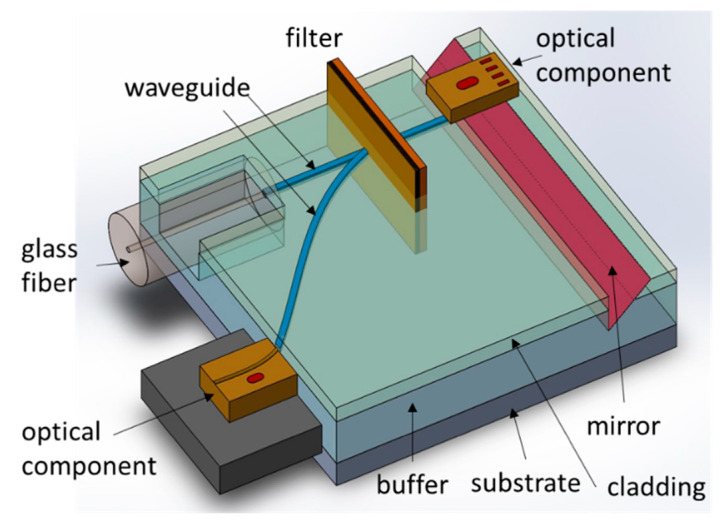
Schematic presentation of an optical microchip on a polymer platform, with dark blue lines depicting polymer optical waveguides.

Generally, two materials are required for the fabrication of a planar optical waveguide. One is the core material with a refractive index of 1.48–1.50, and the other is the cladding material with a refractive index of 1.45 [6,7,10], usually close to the refractive index of fused silica (1.444 at 1550 nm). The required refractive index contrast between the core and the cladding, which can be 0.05, 0.03 or even lower, is determined by the particular application. The only materials commercially available for this purpose (ZPU, ChemOptics, Daejeon, Korea) are mixtures of fluorinated acrylates of proprietary composition and possess rather high TOC of −(1.5–2.2) × 10^−4^ K^−1^ [11]. Therefore, the challenge is to develop alternative materials with lower TOC for passive optical waveguides. Passive optical waveguides are important components of optical microchips based on polymer platforms [12]. A schematic presentation of an optical microchip on a polymer platform is presented in Scheme 1, where dark blue lines depict polymer optical waveguides [13].

A low-refractive index and low optical losses at telecommunication wavelength (ca. 1550 nm) are usually achieved in polymeric materials by increasing the concentration of C–F (C–Cl, C–Br) bonds and by avoiding of O–H and N–H bonds, which show strong absorption in this wavelength region. The refractive indices of core and cladding polymers are tuned by changing the polymer chemical composition. Organic or hybrid polymers are usually used as waveguiding materials [11,14,15,16,17,18]. However, the refractive indices of Ormocer hybrid polymers are usually higher than 1.5 [14,15,17].

A combination of inorganic and organic materials as nanocomposites for waveguiding materials has not been exploited in detail so far. Sufficiently small nanoparticles (NPs), actually smaller than 1/20 of the application wavelength (in our case smaller than ca. 75–80 nm), should produce very weak scattering and, in this way, should not have a significant negative influence on the optical propagation losses.

Firstly, the idea of using inorganic materials originates from their considerably lower TOC, which is caused by their lower thermal expansion coefficient. In addition, some inorganic materials (e.g., MgF_2_, CaF_2_, AlF_3_, SiO_2_) also exhibit low-refractive indices and thus yield the opportunity to reduce the refractive index of the resulting nanocomposite. Therefore, by using inorganic NPs, it would be possible to tune refractive index and TOC in the direction of lower values. For example, it was rather interesting to apply MgF_2_, which possesses the lowest refractive index of 1.37 at 1550 nm, and these NPs were already introduced into polymeric matrices [19,20]. It was demonstrated that they were able to decrease the refractive index of nanocomposites [19]. We recently used this approach, applying both SiO_2_ and MgF_2_ NPs [21,22]. Silica NPs were the evident object in this case, as they were used, along with other oxide NPs (e.g., TiO_2_, ZrO_2_), for holographic nanocomposites based on acrylates mixtures [23,24,25]. TiO_2_ and ZrO_2_ NPs could be used to prepare high-refractive index nanocomposites [26]. In a previous work, we introduced SiO_2_ NPs into a solid polymer matrix [21]. The polymers capable of photochemical cross-linking due to oxirane groups were used in this case. Afterwards, the polymer nanocomposites were prepared; they were cross-linked on a substrate using UV-exposure forming an insoluble layer as a component of an optical waveguide (cladding and core). Since it was only partially successful, this approach led to the formation of cracks for thicker waveguide layers and, therefore, film-building properties should be improved. A recent study with MgF_2_ NPs resulted in an increase in optical losses of polymer layers, probably due to absorption of the organic shell on MgF_2_ NPs around 1550 nm [22]. While the influence of NPs on a refractive index and TOC is theoretically clear [27], the impact of NPs distributed in a polymer matrix on optical propagation losses is not widely studied. Inorganic materials (core of NP) have to possess a positive influence on optical losses, but their organic shell, which is required for the compatibility of NPs with a polymer matrix, might have a negative influence, depending on the shell’s chemical composition [22]. In addition, if NPs are not sufficiently small or if they agglomerate in the matrix, scattering will also increase the optical propagation losses.

Thus, in this work, we studied the impact of SiO_2_ NPs distributed in low-refractive index acrylate monomer mixtures on the fabrication and properties of planar optical waveguides. The monomer formulations applied were custom-made and consisted of acrylates cross-linked via photochemical radical polymerization. The composition of acrylate mixtures was tuned in order to achieve the required refractive index and viscosity for the fabrication of core and cladding for the planar optical waveguide. The objective of this work was to introduce SiO_2_ NPs into these mixtures and to investigate their influence on optical properties of prepared waveguides (refractive index, TOC, and propagation losses). The goal was also to achieve better processability compared to nanocomposites based on solid polymers [21]. SiO_2_ NPs were used for the first time in such formulations for the fabrication of optical waveguides, and, by using the NPs, we tried to improve the properties of waveguides by reducing their TOC with the conservation of their propagation losses.

## 2. Materials and Methods

### 2.1. Materials and Film Preparation

**Scheme 2:**
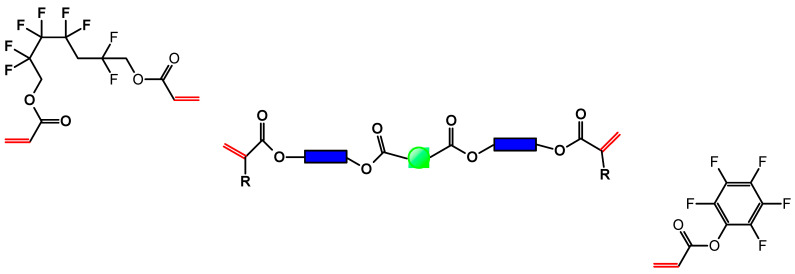
Examples of fluorinated acrylate, diacrylate, and viscous polyester acrylate for the formulation of liquid monomer waveguiding materials.

Highly viscous acrylate oligomers (Scheme 2) were free samples from Sartomer by Arkema Group (Colombes, France) and MIWON Specialty Co. Ltd. (Gyeonggi-do, South Korea). Pentaerythritoltetraacrylate (PETA) was purchased from Sigma-Aldrich (St. Louis, MO, USA). Fluorinated acrylates (typical examples are shown in Scheme 2) were purchased from ABCR (Karlsruhe, Germany), Exfluor (Round Rock, TX, USA), Fluorochem (Hadfield, UK), and P&M-Invest (Moscow, Russian Federation). Proprietary acrylate monomer formulations were prepared by mixing fluorinated acrylates and diacrylates (Scheme 2) with viscous acrylate polyether oligomers in different compositions to achieve the target refractive index and the viscosity suitable for the preparation of 3–20 µm-thick films. Pentafluorophenyl acrylate was used to increase the refractive index while perfluorohexanedioldiacrylate (or dimethacrylate) and fluorinated tetraethyleneglycoldiacrylate were predominantly used to decrease the refractive index. Sartomer CN2036 (Arkema) and HR6060 (Miwon) were used predominantly to increase viscosity and refractive index. Silica NP suspensions MEK-ST, PMA-ST, TOL-ST were purchased from Nissan Chemical Industries Ltd. (Tokyo, Japan). Silica NP dispersions Nanopols and Nanocryls were free samples from Evonik Resource Efficiency GmbH (Essen, Germany). Silica NP dispersion Nanobyk 3605 was a free sample from BYK-Chemie GmbH (Wesel, Germany). The properties of silica NP dispersions are collated in Table 1. 2-Hydroxy-2-methylpropiophenone and 2,4,6-trimethyl-benzoyldiphenylphosphinoxide (both Sigma-Aldrich) were used as radical photoinitiators.

Commercial material for optical waveguide fabrication ZPU 480 (ChemOptics, Daejeon, Korea) was received from C. Zawadzki (Fraunhofer Heinrich-Hertz Institute, Berlin, Germany) in the framework of the PolyPhotonics Project.

Monomers were mixed under slight heating and magnetic stirring. Then, NPs were introduced into monomer mixtures. An excess of solvent in the case of the NPs dispersed in solvents (Table 1) was removed by heating them gently to achieve the viscosity suitable for layers preparation (control by layer thickness measured by m-line spectroscopy). In case of a highly volatile solvent, such as 2-butanone, after solvent removal, another solvent (cyclopentanone) more suitable for spin-coating was added.

Thin layers (3–20 µm) of monomer formulations and nanocomposites were prepared by spin-coating using a P6700 spin-coater (Specialty Coating Systems, Indianapolis, IN, USA) on Si wafers or fused silica substrates. The films were exposed to UV light using an UV-H 200 mercury lamp (Panacol-Elosol GmbH, Steinbach/Taunus, Germany) and then annealed at 160 °C. The films exhibited good adhesion properties on both Si and fused silica substrates and usually did not require an adhesion promoter.

### 2.2. Measurements

Optical properties of polymer films (refractive index and thickness, TOC, optical propagation losses) were measured using m-line spectroscopy performed on a Metricon Model 2010/M Prism Coupler System (Metricon Inc., Pennington, NJ, USA) with polymer films on Si wafers and fused silica substrate. At first, the technique was routinely used to tune and control refractive index and film thickness. Then, for already optimized formulations, dependencies of the refraction index on temperature were measured. This allowed us to determine TOC and glass transition temperature. Optical losses of the planar waveguides were measured using a technique involving the measurement of transmitted and scattered light intensity as a function of propagation distance along the waveguide [28]. The values of optical losses were calculated in Metricon software using exponential fitting of the scattered light intensity vs. distance curve. In some cases, fitting to valleys was used, where fitting was obtained only on chosen areas of the curve. This measuring technique has been also used recently for the characterization of other polymer optical waveguides [17,27,28,29,30,31,32].

For transmission electron microscopy (TEM) investigations the polymer film was embedded in methacrylate and polymerized by UV-radiation for 4 h. Ultrathin cuts (thickness ~60 nm) of the sample were prepared with an ultra-microtome Leica EM UC7 (Leica Microsystems, Wetzlar, Germany) using a diamond knife. Micrographs were taken with a transmission electron microscope JEM-1400 Plus (Jeol Ltd., Tokyo, Japan) at an acceleration voltage of 120 kV and processed with a digital imaging software ICW (Boerder electronics).

## 3. Results and Discussion

Commercial SiO_2_ NPs from Nissan, Evonik, and BYK (Table 1) were used for the direct introduction of SiO_2_ NPs into the monomer formulation and, thus, further into the polymer matrix after photochemical cross-linking. NPs from Nissan and Nanopol NPs from Evonik are NP dispersions in solvents of different volatility. From this point of view, the solvents in MEK-ST and Nanopol C784 are more volatile (Table 1) and therefore are easier to remove if required. Other nanomaterials (Nanocryls and Nanobyk) contain acrylate monomers without fluorine and, therefore, could increase optical losses due to a high concentration of C-H bonds in the acrylate dispersion media (e.g., hexanedioldiacrylate).

### 3.1. Nanocomposites Based on NP Dispersions in Solvents

**Figure 1:**
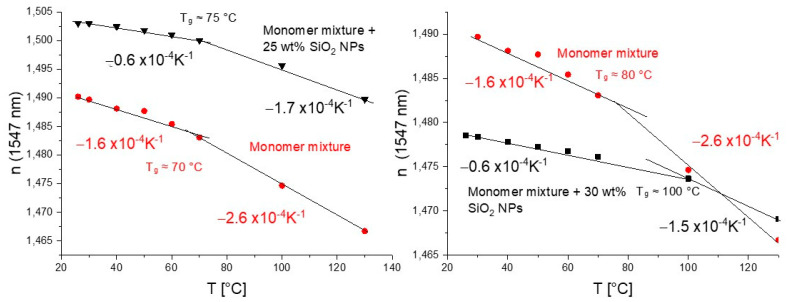
Temperature dependence of refractive index (numbers are thermo-optic coefficients (TOCs) determined as slopes of lines): **left**—core acrylate mixture (n *≈* 1.5) containing 25 wt% of SiO_2_ NPs (from Nissan MEK-ST) and base core mixture (n *≈* 1.49) in red; **right**—core acrylate mixture (n *≈* 1.48) containing 30 wt% of SiO_2_ NP (Nanopol C784) and base core mixture (n *≈* 1.49) in red.

The experiments with NPs from Nissan were focused on using MEK-ST suspension because, in this case, 2-butanone could be easier removed and substituted with another solvent more suitable for spin-coating (cyclopentanone). Both core (n = 1.50 and n = 1.48) and cladding (n = 1.45) formulations were successfully prepared, and the concentration of SiO_2_ NPs varied from 10 to 30 wt%. Generally, 2-butanone, which is not a particularly good solvent for spin-coating due to its rather low boiling point (80 °C), was substituted completely or partially with cyclopentanone (b.p. 131 °C). Most of these mixtures, where the monomer composition was also tuned to achieve the required refractive index, produced optical layers of sufficient quality and thickness for complete optical characterization (refractive index, thickness, TOC, glass transition temperature, T_g_, and optical propagation losses). Photochemically cured polymeric nanomaterials exhibited a relatively low TOC of −(0.6–1.0) × 10^−4^ K^−1^, depending on the composition. These values are much lower than the values for the original acrylate mixture (−(1.3–1.5) × 10^−4^ K^−1^) and commercial ZPU materials (−(1.5–2.2) × 10^−4^ K^−1^) [11]. An example of a measurement for a material with low TOC is shown in Figure 1, left.

The measurement of temperature dependence of refractive index also allows the determination of T_g_ of the polymer film that is produced after photochemical cross-linking. This measurement method, along with temperature-dependent spectroscopic ellipsometry [21,33], allows for the measuring of T_g_ in thin films, while other techniques (DSC, DMA) are suitable only for bulk materials. The advantages of m-line spectroscopy are fast measurement and the fact that T_g_ is determined in the form (thin film) exactly used for the application. Thin film T_g_ can differ significantly from bulk T_g_ [33]. The glass transition is usually manifested by the kick in the refractive index-temperature dependence (an increase in TOC, see Figure 1). No influence on T_g_ was observed in this case. This means that SiO_2_ NPs show little impact on T_g_ of nanocomposites and that, in fact, T_g_ is determined only by monomer composition. This was not surprising as it was demonstrated for two different polymer matrices experimentally and theoretically [34] that the introduction of SiO_2_ NPs into a polymer matrix increases T_g_ by 10 °C only.

**Figure 2:**
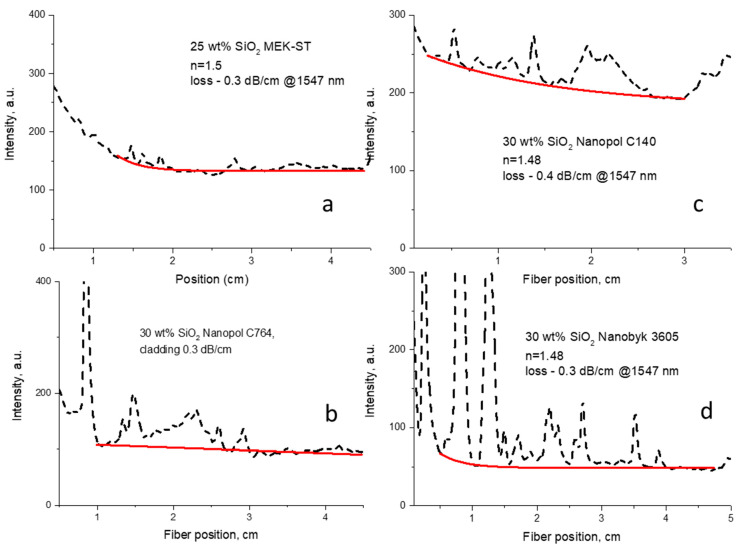
Examples of optical propagation losses for polymer nanocomposites with SiO_2_ NPs from different sources: solvent dispersions, (**a**) core material (n ≈ 1.5) containing 25 wt% SiO_2_ (MEK-ST); (**b**) cladding material containing 30 wt% SiO_2_ (Nanopol C764); acrylate dispersions, (**c**) core material (n ≈ 1.48) containing 30 wt% SiO_2_ (Nanocryl C140); (**d**) core material (n ≈ 1.48) containing 30 wt% SiO_2_ (Nanobyk 3605); values of losses are indicated (red lines are exponential fits).

The values of optical propagation losses varied in this case from 0.2 to 1.0 dB/cm at 1547 nm with the best samples being comparable to commercial ZPU material (0.35 dB/cm [13]) or Ormocer-type materials (0.5–0.7 dB/cm at 1547 nm [14,17]). An example of the optical loss measurement for nanocomposites with MEK-ST is shown in Figure 2a.

**Figure 3:**
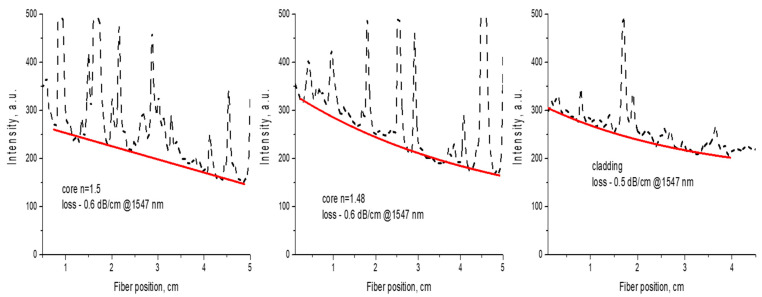
Examples of optical propagation losses for waveguiding polymers from fluorinated acrylate formulations (base materials for nanocomposites): core n = 1.50 and n = 1.48, cladding (red lines are exponential fits).

The values are also similar to those obtained for original acrylate monomer mixtures used for the fabrication of nanocomposites (Figure 3).

Similar results were obtained using SiO_2_ NP solvent dispersions from Evonik (Nanopol C764, C784). For different mixtures (core and cladding) containing 20–40 wt% of NPs and different fluorinated and non-fluorinated acrylates, the value of TOC was generally below −1.0 × 10^−4^ K^−1^, with lowest value up to −0.6 × 10^−4^ K^−1^. An example of the measurement of the formulation with low TOC using Nanopol C784 is shown in Figure 1, right. Thus, the effect of lowering of TOC caused by inorganic material is also clearly demonstrated in this case (Nanopol NP dispersions). The influence of NPs on T_g_ was not high in this case (increase by 20 °C maximum, Figure 1, right). This also corresponds to the data for other nanocomposites [30]. The values of propagation optical losses were also between 0.2 and 1.0 dB/cm at 1547 nm. This demonstrates that the best values are similar to commercial ZPU material (0.35 dB/cm [11]), Ormocer-type materials (0.5–0.7 dB/cm [14,17]), and original acrylate mixtures (Figure 3). An example of optical loss measurement is shown in Figure 2b for cladding material. It is important to emphasize that, by using SiO_2_ NP dispersions in organic solvents, it was possible to prepare both core and cladding nanomaterials.

### 3.2. Nanocomposites Based on NP Dispersions in Acrylates

Less conclusive were the results obtained on the basis of SiO_2_ NP dispersions in acrylates (Nanocryls and Nanobyk). NP-monomer formulations with an NP concentration of 20–30 wt% were also tested in this case. It should be mentioned that, in this case, there was a simultaneous increase in inorganic content and in the concentration of C-H bonds, due to an addition of non-fluorinated acrylate (e.g., hexanedioldiacrylate) that served as dispersion medium. In any case, the TOC values of –(0.8–1.5) × 10^−4^ K^−1^ were registered and such values of TOC did not show a clear difference from the base materials, or this difference was relatively small (the values of TOC for the original acrylate mixture were −(1.3–1.5) × 10^−4^ K^−1^). Optical propagation losses were between 0.2 and 1.3 dB/cm at 1547 nm. Even in this case, the best samples exhibited low values of optical losses (Figure 2c,d), despite the possible additional contribution to optical losses from non-fluorinated acrylates used as dispersing media in Nanocryl and Nanobyk dispersions. 

**Figure 4:**
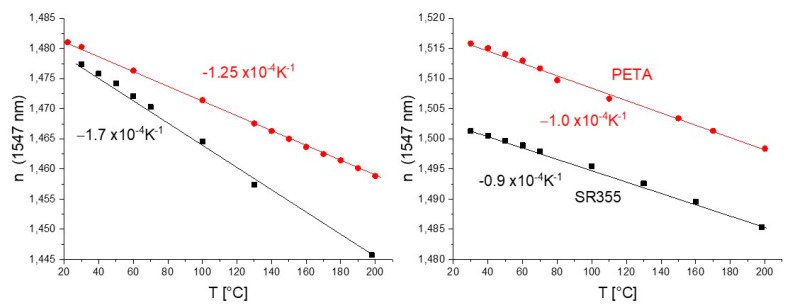
Temperature dependence of refractive index (numbers are TOCs determined as slopes of lines): **left**—core acrylate mixture (n *≈* 1.48) containing SiO_2_ NPs (from Nanopol C764, 20 wt% of SiO_2_, in red from Nanocryl C140, 30 wt% of SiO_2_); **right**—highly cross-linking monomers: Pentaerythritoltetraacrylate (PETA) and Sartomer SR355.

The T_g_ values for the nanocomposite materials studied were usually relatively low, around 100 °C (data not shown), similar to materials based on SiO_2_ dispersions in solutions (Figure 1). However, some examples of NP-acrylate mixtures based on NP dispersions in acrylates exhibited relatively high TOC (−(1.2–1.7) × 10^−4^ K^−1^), but also an absence of glass transition in the measured temperature range (25–200 °C) (Figure 4). By analyzing the results, it was found that similar behavior was observed for pure strongly cross-linking monomers (PETA, Sartomer SR355) (Figure 4) and commercial ZPU material [13]. It seems that the synergic effect of NPs and cross-linking multifunctional acrylates such as hexanedioldiacrylate, Sartomer CN2036 or Miramer HR6060, which were used for the formulation of mixture or as dispersing media of NP dispersions, leads to such a degree of cross-linking that glass transition is not observed at least up to 200 °C. The concentration of these cross-linking acrylates is higher in these cases compared to formulations based on NP dispersions in solvents, due to the additional contribution of acrylate dispersion media in Nanocryl and Nanobyk. 

It is clear that SiO_2_ NPs are able to affect thermo-optical properties of resulting polymers, reducing TOC (as expected), and also suppressing glass transition in some cases. However, nanomaterials with lower TOC (in our case, based on NPs dispersed in solvents) are of more interest for application in passive optical waveguides. In addition, taking into account the somewhat lower values of optical losses for these formulations and the possibility to formulate both core and cladding materials, it can be concluded that nanocomposites based on SiO_2_ NP dispersions in solvents are preferable materials.

### 3.3. Comparison of Optical Propagation Losses

The magnitude of optical losses is rather important for the practical application in planar waveguides, and the target for the fabrication of optical microchips on polymer platforms is to keep optical losses below 0.6 dB/cm at 1547 nm [10,11,12]. Basis formulations of fluorinated acrylate materials used in this study exhibited such values in the best examples (Figure 3).

**Figure 5:**
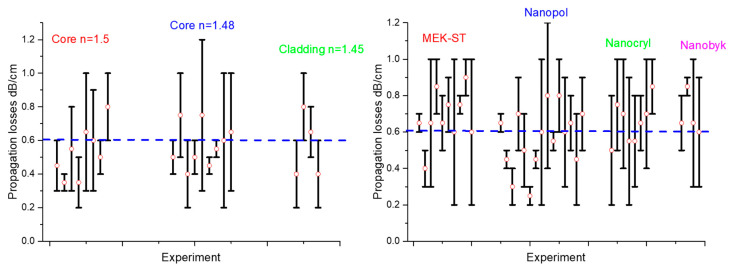
Overview of the loss measurement for basis acrylate mixtures (**left**) and for all studied in Scheme 2. NPs from different sources (**right**). Value spreading corresponds to the measurements using different samples of the same material and measurements along different optical paths in the same sample.

If we plot the results of optical loss measurements obtained from different acrylate formulations and different samples, we see considerable deviation in the measurements (Figure 5, left). However, despite deviations, the median value of the measurement is close to 0.6 dB/cm at 1547 nm, which is quite acceptable. The deviation can be explained by defects in the layers caused by the preparation, as measurements were carried out using different optical paths in the same sample, by rotating the sample over the coupling point, by coupling the sample at different spots and by using different samples. The quality of layers also depends on the quality of the clean-room atmosphere.

**Figure 6:**
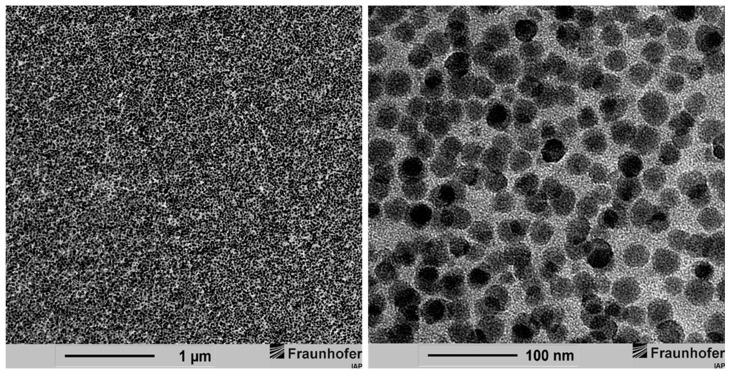
Transmission electron microscopy (TEM) measurement of the film prepared with SiO_2_ NP dispersion Nanopol C 764 (30 wt% of SiO_2_) at different magnifications.

The quality of the films prepared in this work was also confirmed by transmission electron microscopy (TEM), and an example of TEM measurement is presented in Figure 6. These measurements show that SiO_2_ NPs are evenly distributed in the polymer matrix without formation of the aggregates, which might affect optical propagation losses. The size of NPs seems to be around 15–30 nm, which corresponds well to the data of the manufacturer (20 nm, see Table 1). It would be difficult to expect better distribution by such high NP concentration (30 wt%). Similar results were obtained in a recent publication [35] with larger SiO_2_ NPs in PMMA matrix.

A similar diagram was plotted for all nanocomposite samples (Figure 5, right). It also shows a similar trend with the median value of around 0.6 dB/cm. It demonstrates that waveguides with propagation losses acceptable for the application level could also be prepared by using nanocomposite formulations. This leads to the possibility of waveguide fabrication completely from nanocomposites and by using SiO_2_ NP dispersed in solvent; it would be possible to prepare a complete waveguide using NPs from the same source.

The fact that polymers have the potential to be used in harsh environmental conditions in optical applications is demonstrated by the optical glass fibers. A variant of the optical glass fibers is the so-called “polymer-clad” fiber, which has an optical cladding made of a cross-linked polymer, which has high thermal stability and high resistance against high optical power. These glass fibers, for example, are used for the transmission of laser radiation in material-processing lasers and surgical lasers. Laser powers of up to 100 W are transmitted in these cases. In addition, the fibers for medical applications can be autoclaved several times with hot steam [36].

**Figure 7:**
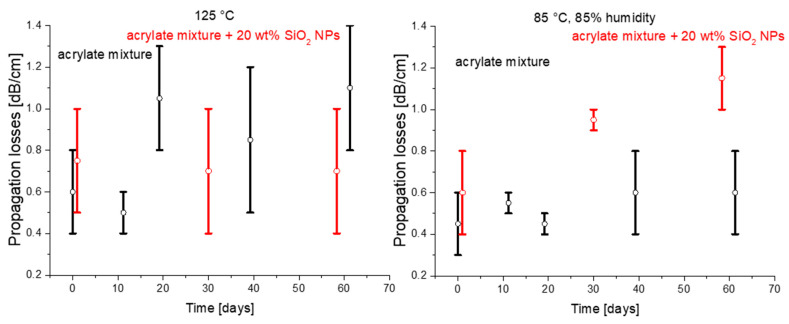
Optical loss investigation of the samples from basis material and nanocomposite containing 20 wt% SiO_2_ (Nanobyk 3605) in dependence of climate conditions (temperature—**left**, temperature and humidity—**right**).

Due to the high stability of the C-F bonds, fluorinated polymers typically show an increased thermal and media stability in comparison to their non-fluorinated counterparts [37,38]. Additional stability could be reached by introducing inorganic materials, namely, nanocomposites. Therefore, the impact under harsh environmental conditions (heat and high humidity) on the optical losses has also been investigated. The comparison of original acrylate mixtures and nanocomposites based on them in terms of environmental stability is shown in Figure 7. It seems that if, at a high temperature (125 °C) nanocomposites show better performance (Figure 7, left), at high humidity and high temperature conditions (Figure 7, right) nanocomposites clearly exhibit inferior behavior. Tentatively, it could be linked to possible absorption of water vapors by silica and a decrease in the concentration of C-F bonds.

## 4. Conclusions

Successful introduction of SiO_2_ NPs into optical polymer layers without additional scattering was demonstrated. The nanocomposite formulations were prepared by direct introduction of SiO_2_ NPs from different sources, dispersed in solvents or acrylate monomers, into fluorinated acrylate mixtures. It was demonstrated that core and cladding materials with sufficiently good film building properties can be obtained in this way, yielding the possibility for the preparation of complete waveguide from nanocomposites. The results also exhibit a significant decrease in the thermo-optic coefficient of the material, without a noticeable change in propagation optical losses in planar waveguides. Some materials exhibited thermally stable behavior with glass transition temperatures above 200 °C. Better stability at a temperature of 125 °C was also observed.

## Figures and Tables

**Table 1:** Characteristics of SiO_2_ NP sources.

Material	Manufacturer	NP Diameter [nm]	Solvent/Carrier
MEK-ST	Nissan	15–20	2-Butanone
PMA-ST	Nissan	15–20	Methoxypropylacetate acetate
TOL-ST	Nissan	15–20	Toluene
Nanocryl A225	Evonik	20	Trimethylolpropantriacrylate
Nanocryl A235	Evonik	20	Alkoxylated (4) pentaerythritoltetraacrylate
Nanocryl C140	Evonik	20	Hexanedioldiacrylate
Nanocryl C145	Evonik	20	Tripropyleneglycoldiacrylate
Nanocryl C150	Evonik	20	Trimethylolpropanetriacrylate
Nanocryl C165	Evonik	20	Alkoxylated (4) pentaerythritoltetraacrylate
Nanopol C764	Evonik	20	Methoxypropylacetate
Nanopol C784	Evonik	20	*n*-Butylacetate
Nanobyk 3605	BYK	20	Hexanedioldiacrylate

## Data Availability

Data is contained within the article.

## References

[1] Matsuura T., Ando S., Sasaki S., Yamamoto F. (1994). Polyimides Derived from 2,2′-Bis(trifluoromethyl)-4,4′-diaminobiphenyl. 4. Optical Properties of Fluorinated Polyimides for Optoelectronic Components. Macromolecules.

[2] Fischbeck G., Moosburger R., Kosttzewa C., Aeben A., Petermann K. (1997). Singlemode optical waveguides using a high temperature stable poylmer with low losses in the 1.55 µm range. Electron. Lett..

[3] Yoshimura R., Hikoto M., Tomaru S., Imamura S. (1998). Low-loss polymeric optical waveguides fabricated with deuterated polyfluoromethacrylate. J. Lightwave Technol..

[4] Lee H.J., Lee M.H., Oh M.C., Ahn J.H., Han S.G. (1999). Crosslinkable polymers for optical waveguide devices. II. Fluorinated ether ketone oligomers bearing ethynyl group at the chain end. J. Polym. Sci. A Polym. Chem..

[5] Kang J.W., Kim J.P., Lee W.Y., Kim J.S., Lee J.S., Kim J.J. (2001). Low-loss fluorinated poly(arylene ether sulfide) waveguides with high thermal stability. J. Lightwave Technol..

[6] Oh M.-C., Kim K.-J., Chu W.-S., Kim J.-W., Seo J.-K., Noh Y.-O., Lee H.-J. (2011). Integrated photonic devices incorporating low-loss fluorinated polymer materials. Polymers.

[7] Keil N., Yao H.H., Zawadzki C., Beyer F., Radmer O., Bauer M., Dreyer C., Han S.-G., Lee H.-J. Polarisation and temperature behaviour of AWGs based on commercial low-loss polymers. In Proceedings of the International Cooperative of Plastic Optical Fibres -ICPOF: 13th International Plastic Optical Fibres Conference.

[8] Dreyer C., Schneider J., Göcks K., Beuster B., Bauer M., Keil N., Yao H., Zawadzki C. (2003). New reactive polymeric systems for use as waveguide materials in integrated optics. Macromol. Symp..

[9] Ziemann O., Krauser J., Zamzow P.E., Daum W. (2008). POF Handbook, Optical Short Range Transmission Systems.

[10] Zhang Z., Mettbach N., Zawadzki C., Wang J., Schmidt D., Brinker W., Grote N., Schell M., Keil N. (2011). Polymer-based photonic toolbox: Passive components, hybrid integration and polarisation control. IET Optoelectron..

[11] Waveguide Resin. http://www.chemoptics.co.kr/eng/sub/sub01_01.php?cat_no=30.

[12] Zawadzki C., Schirmer M. (2018). Der Wellenleiter-Chip—Photonische Bauelemente aus Polymeren. Photonik.

[13] PolyPhotonics Berlin. http://www.polyphotonics-berlin.de/projekte/.

[14] Buestrich R., Kahlenberg F., Popall M., Danberg P., Müller-Fiedler R., Rösch O. (2001). ORMOCER^®^s for Optical Interconnection Technology. J. Sol-Gel Sci. Technol..

[15] Houbertz R., Domann G., Cronauer C., Schmitt A., Martin H., Park J.-U., Fröhlich L., Buestrich R., Popall M., Streppel U. (2003). Inorganic–organic hybrid materials for application in optical devices. Thin Solid Films.

[16] Prajzler V., Neruda M., Jasek P., Nekvindova P. (2019). The properties of free-standing epoxy polymer multi-mode optical waveguides. Microsyst. Technol..

[17] Prajzler V., Jasek P., Nekvindova P. (2019). Inorganic–organic hybrid polymer optical planar waveguides for micro-opto-electro-mechanical systems (MOEMS). Microsyst. Technol..

[18] Ishigure T., Yoshida S., Yasuhara K., Suganuma D. Low-loss single-mode polymer optical waveguide at 1550-nm wavelength compatible with silicon photonics. Proceedings of the 2015 IEEE 65th Electronic Components and Technology Conference (ECTC).

[19] Noack J., Fritz C., Flügel C., Hemmann F., Gläsel H.-J., Kahle O., Dreyer C., Bauer M., Kemnitz E. (2013). Metal fluoride-based transparent nanocomposites with low refractive indices. Dalton Trans..

[20] Noack J., Schmidt L., Gläsel H.-J., Bauer M., Kemnitz E. (2011). Inorganic–organic nanocomposites based on sol–gel derived magnesium fluoride. Nanoscale.

[21] Goldenberg L.M., Köhler M., Kahle O., Dreyer C. (2020). Impact of inorganic nanoparticles on optical properties of low refractive index waveguiding polymers. Opt. Mater. Express.

[22] Goldenberg L.M., Köhler M., Dreyer C., Krahl T., Kemnitz E. (2021). Optical Nanocomposites Containing Low Refractive Index MgF_2_ Nanoparticles. Eur. Phys. J. Appl. Phys..

[23] Sakhno O.V., Goldenberg L.M., Stumpe J., Smirnova T.N. (2009). Effective volume holographic structures based on organic–inorganic photopolymer nanocomposites. J. Opt. A Pure Appl. Opt..

[24] Tomita Y., Hata E., Momose K., Takayama S., Liu X., Chikama K., Klepp J., Pruner C., Fally M. (2016). Photopolymerizable nanocomposite photonic materials and their holographic applications in light and neutron optics. J. Mod. Opt..

[25] Sunchez C., Escuti M.J., van Heesch C., Bastiaansen C.W.M., Broer D.J., Loos J., Nussbaumer R. (2005). TiO_2_ Nanoparticle-Photopolymer Composites for Volume Holographic Recording. Adv. Funct. Mater..

[26] Tsai C.-M., Hsu S.-H., Ho C.-C., Tu Y.-C., Tsai H.-C., Wang C.-A., Su W.-F. (2014). High refractive index transparent nanocomposites prepared by in situ polymerization. J. Mater. Chem. C.

[27] Zou H., Wu S., Shen J. (2008). Polymer/Silica Nanocomposites: Preparation, Characterization, Properties, and Applications. Chem. Rev..

[28] Nourschargh N., Starr E.M., Fox N.I., Jones S.G. (1985). Simple Technique for Measuring Attenuation of Integrated Optical Waveguides. Electron. Lett..

[29] Prajzler V., Nekvindova P., Hyps P., Jerabek V. (2015). Properties of the Optical Planar Polymer Waveguides Deposited on Printed Circuit Boards. Opt. Commun..

[30] Prajzler V., Hyps P., Mastera R., Nekvindova P. (2016). Properties of Siloxane Based Optical Waveguides Deposited on Transparent Paper and Foil. Opt. Mater..

[31] Prajzler V., Neruda M., Nekvindova P. (2018). Flexible multimode polydimethyl-diphenylsiloxane optical planar waveguides. J. Mater. Sci. Mater. Electron..

[32] Chiu J.-J., Perng T.P. (2008). The passive optical properties of a silicon nanoparticle-embedded benzocyclobutene polymer waveguide. Nanotechnology.

[33] Bittrich E., Windrich D., Martens L., Bittrich L., Häussler L., Eichhorn K.-J. (2017). Determination of the glass transition temperature in thin polymeric films used for microelectronic packaging by temperature-dependent spectroscopic ellipsometry. Polym. Test..

[34] Ahn S.I., Ohk S.W., Kim J.H., Zin W.-C. (2009). Glass Transition Temperature of Polymer Nanocomposites: Prediction from the Continuous-Multilayer Mode. J. Polym. Sci. B.

[35] Boucher V.M., Cangialosi D., Alegria A., Colmenero J., Gonzalez-Irun J., Liz-Marzan L.M. (2010). Accelerated physical aging in PMMA/silica nanocomposites. Soft Matter.

[36] LEONI. https://www.leoni-fiber-optics.com/en/applications/healthcare/.

[37] Hougham G.G., Cassidy P.E., Johns K., Davidson T. (2002). Fluoropolymers 1: Synthesis.

[38] Dreyer C., Schrader S., Yao H. (2020). Fluorinated thermosetting resins for photonic applications. Fascinating Fluoropolymers and Their Applications.

